# SALT OVERLY SENSITIVE 1 Na^+^/H^+^ Exchanger Operates in Mature Root Zone and Is a Major Contributor to Root Na^+^ Exclusion During Shoot‐to‐Root Na^+^ Recirculation

**DOI:** 10.1111/pce.70317

**Published:** 2025-12-12

**Authors:** Tomoki Nagata, Ryohei Sugita, Takaaki Ogura, Mio Nagoya, Natsuko I. Kobayashi, Muhammad B. Gill, Lana Shabala, Tomoko M. Nakanishi, Sergey Shabala, Keitaro Tanoi

**Affiliations:** ^1^ Graduate School of Agricultural and Life Sciences The University of Tokyo Bunkyo‐ku Tokyo Japan; ^2^ Radioisotope Research Center Nagoya University Nagoya Aichi Japan; ^3^ Tasmanian Institute of Agriculture, College of Science and Engineering University of Tasmania Hobart Tasmania Australia; ^4^ International Research Centre for Environmental Membrane Biology Foshan University Foshan China; ^5^ School of Biological Science University of Western Australia Perth Western Australia Australia; ^6^ Fukushima Institute for Research, Education and Innovation Fukushima Japan

**Keywords:** MIFE, phloem transport, radioisotope, salinity, sodium, SOS1, xylem transport

## Abstract

The Na^+^/H^+^ antiporter SALT OVERLY SENSITIVE 1 (SOS1) is a key component of Na^+^ exclusion and plant salt tolerance. Although previous studies have suggested that SOS1 functions in both the root apex and mature root zone, their contributions remain unclear due to limited methodological resolution and originated mostly from transcriptional analysis. Here, we performed isotopic tracing techniques to visualize and quantify Na^+^ exclusion. Real‐time imaging of shoot‐applied ^22^Na^+^ showed that ^22^Na^+^ gradually disappeared from roots in wild‐type (WT) plants, whereas it did not in *sos1* mutants. To confirm that this reduction reflected active Na^+^ exclusion to the rhizosphere, we used the Microelectrode Ion Flux Estimation, which revealed significant Na^+^ efflux at the mature root zone of WT plants following shoot Na^+^ application, while no such efflux was observed at the root apex or in either root zone of *sos1* mutants. Further quantification using a radioisotope‐based method showed that approximately 90%–95% of Na^+^ derived from both the phloem and xylem was excluded from WT roots, primarily via SOS1, with the mature root zone identified as the major contributor. This study provides visual and quantitative evidence for the crucial contribution of SOS1 to Na^+^ exclusion in the mature root.

## Summary statement

This manuscript uses radiotracer and MIFE techniques to visualize and quantify Na^+^ dynamics in Arabidopsis roots. We demonstrate for the first time that SOS1 Na^+^/H^+^ antiporter contributes to ~95%–98% of Na^+^ exclusion in the mature root zone, where phloem‐derived Na^+^ is excluded to the rhizosphere. The study provides visual and quantitative evidence for SOS1's dominant role in Na^+^ recirculation and exclusion under salt stress.

## 1 Introduction

High salinity impairs plant growth mainly by causing sodium ion (Na^+^) accumulation, which disrupts enzymatic activities and cellular homeostasis (Shabala and Cuin 2008; Zhao et al. 2020). To mitigate Na^+^ toxicity, plants have evolved several strategies, among which Na^+^ exclusion via plasma membrane transporters is particularly crucial. The Na^+^/H^+^ antiporter SALT OVERLY SENSITIVE 1 (SOS1) is the dominant transporter mediating cytosolic Na^+^ exclusion, which confers salt tolerance across diverse species, including Arabidopsis, rice, tomato and wheat (Cuin et al. 2011; El Mahi et al. 2019; Olías et al. 2009; Shi et al. 2000, 2002). SOS1 activity is primarily regulated by the SOS3 (a calcium‐binding protein, CBL4) and SOS2 (a protein kinase, CIPK24) complex under salinity (Quintero et al. 2011), with additional regulation by CBL10‐CIPK8 complex (Yin et al. 2020) and CBL8‐CIPK24 complex (Steinhorst et al. 2022). Although SOS1 is primarily recognized for excluding Na^+^ from the cytosol to the extracellular space, recent studies have indicated that SOS1 also contributes to vacuolar Na^+^ sequestration from the cytosol in the meristem under salinity conditions (Ramakrishna et al. 2025).

Despite its well‐established essential role in salt tolerance, the spatial expression pattern and precise functional sites of SOS1‐mediated Na^+^ exclusion in roots remain unclear. In Arabidopsis, SOS1 has been reported to localize predominantly in the pericycle and root tip epidermis (Shi et al. 2002, based on GUS staining), whereas other studies have shown a broader expression pattern throughout the root using microarray analysis (Brady et al. 2007) and single‐cell RNA‐Seq (Ryu et al. 2019). Although this discrepancy persists, the prevailing view suggests that SOS1‐mediated Na^+^ exclusion occurs mainly at the root apex (Britto and Kronzucker 2015; Hamam et al. 2016; Van Zelm et al. 2020). Numerous studies have also been conducted based on this premise (Li et al. 2023; Song et al. 2025). However, roots exclude approximately 97%–98% of incoming Na^+^ under salt stress (Munns et al. 2020), and the root apex accounts for only a small fraction of the total root volume. Given this, recent experiments using radioisotopes, which allow spatial separation and pathway‐specific analysis of ion transport, have revealed that SOS1 is also involved in the exclusion of xylem‐derived Na^+^ in the mature root zone (Nagata et al. 2023), although its contribution remains unclear. Collectively, these findings indicate that SOS1 mediates Na^+^ exclusion throughout the root system.

It is known that Na^+^ reaching the leaves can be transported back to the root via the phloem—a process called ‘Na^+^ recirculation’—which is essential for plant Na^+^ tolerance (Matsushita and Matoh 1991; Alfocea et al. 2000) and is mainly mediated by HKT1s (Berthomieu et al. 2003). However, little is known about the fate of Na^+^ after it has been returned to the root, and it remains unclear where and how this phloem‐derived Na^+^ is distributed and excluded within the root system. In particular, understanding how different root regions contribute to SOS1‐mediated Na^+^ exclusion remains a major challenge, as these processes have not yet been quantified or visualized in vivo.

To address these unresolved questions, we employed an integrative approach combining three complementary techniques. First, Real‐Time Radioisotope Imaging (RRIS) was used to visualize the movement of ^22^Na^+^ in intact Arabidopsis plants. Second, the Microelectrode Ion Flux Estimation (MIFE) technique was applied to measure localized Na^+^ efflux at the surface of different root zones. Third, the Air Gap Gel System with radioisotopes enabled quantitative analysis of both the phloem‐derived and xylem‐derived Na^+^ exclusion from various root regions. The primary aim of this study is to quantitatively reveal the actual transport and exclusion of Na^+^ during Na^+^ recirculation in Arabidopsis roots. In addition, we seek to clarify the spatial and quantitative contributions of distinct root zones to SOS1‐mediated Na^+^ exclusion in Arabidopsis, thereby enhancing our understanding of how Na^+^ exclusion is coordinated along the root for comprehensive whole‐plant Na^+^ regulation.

## 2 Results

### 2.1 Real‐Time Radioisotope Imaging Revealed SOS1 Dependent Removal of Phloem‐Derived Na^+^ From the Root

**Figure 1:**
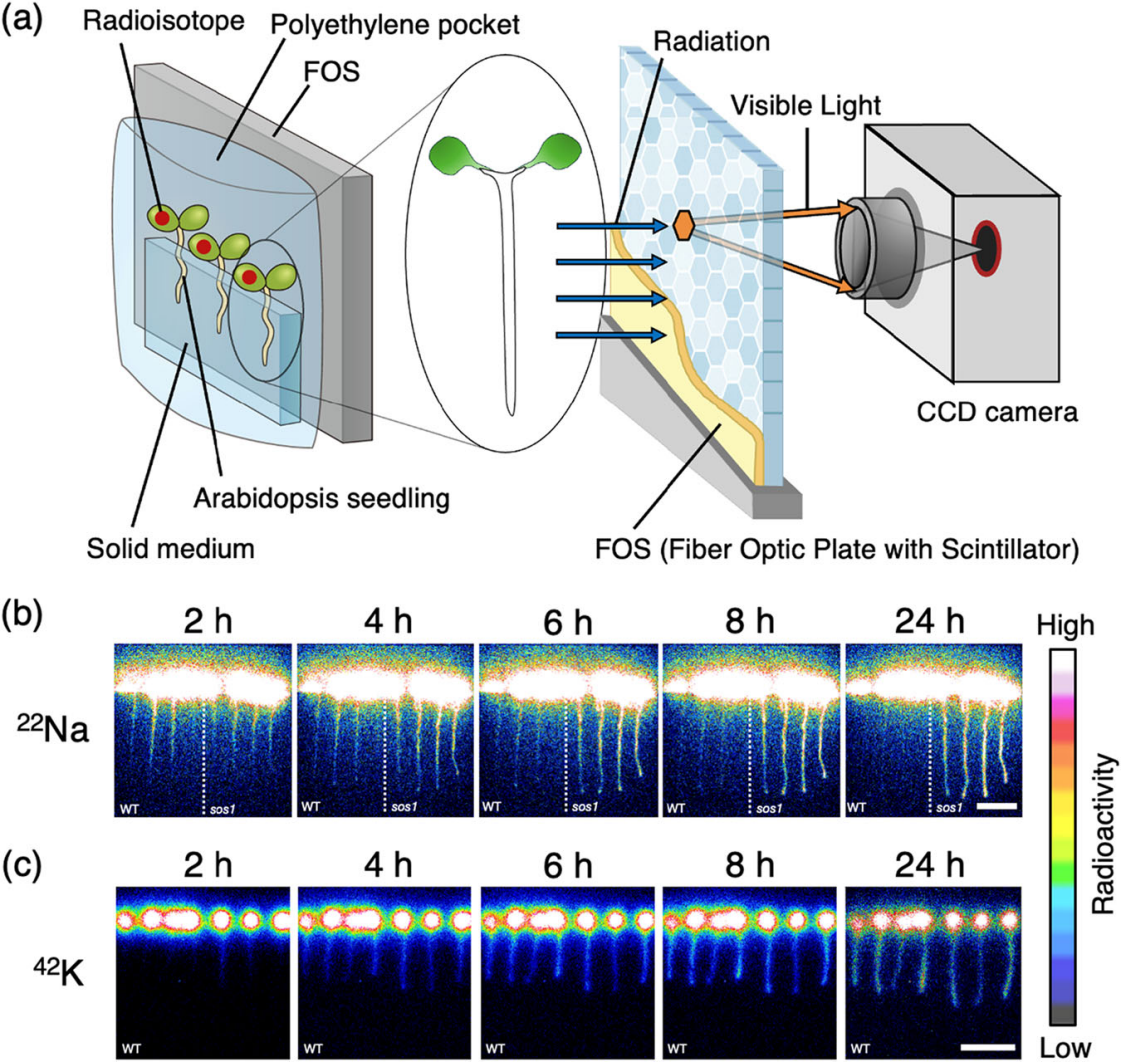
Radioisotope live imaging of solutes in roots. (a) Schematic illustrating the application of radioisotopes to plants using the Real‐Time Radioisotope Imaging System (RRIS). Seven‐day‐old Arabidopsis seedlings were arranged on a solid medium, and radioisotopes were applied to the leaves in solution form. To generate radioisotope images, the radiation emitted from the radioisotopes was converted into visible light and subsequently captured by a CCD camera. (b) RRIS images of radioactive sodium ion (^22^Na^+^) in Arabidopsis WT plants and *sos1* mutants. (c) RRIS images of radioactive potassium ion (^42^K^+^) in WT plants. Scale bars in all images = 20 mm.

First, we investigated the time course of Na^+^ transport using the RRIS (Kanno et al. 2007; Sugita et al. 2014). To obtain clear autoradiograms of ^22^Na in living Arabidopsis roots, we applied ^22^Na^+^ to a leaf and traced phloem‐derived Na^+^ into the root (Figure 1a). In both wild‐type (WT) and *sos1* plants, root ^22^Na signals were detected a few hours after foliar application of ^22^Na^+^. Notably, while the signal intensity decreased in WT roots over time, it remained elevated in *sos1* roots (Figure 1b, Supporting Information S1: Figure S1, Video S1). To confirm whether this removal is specific to Na^+^, we performed RRIS imaging using other ions, including the radioactive potassium isotope ^42^K^+^. Unlike Na^+^, signals of other ions, including ^42^K, remained in WT roots or shoots (Figure 1c, Supporting Information S1: Figure S2, Video S2), suggesting that the removal mechanism observed is specific to Na^+^.

### 2.2 Ion Flux Measurement Detected SOS1‐Mediated Exclusion of Phloem‐Derived Na^+^ From the Mature Root Zone Into the Rhizosphere

**Figure 2:**
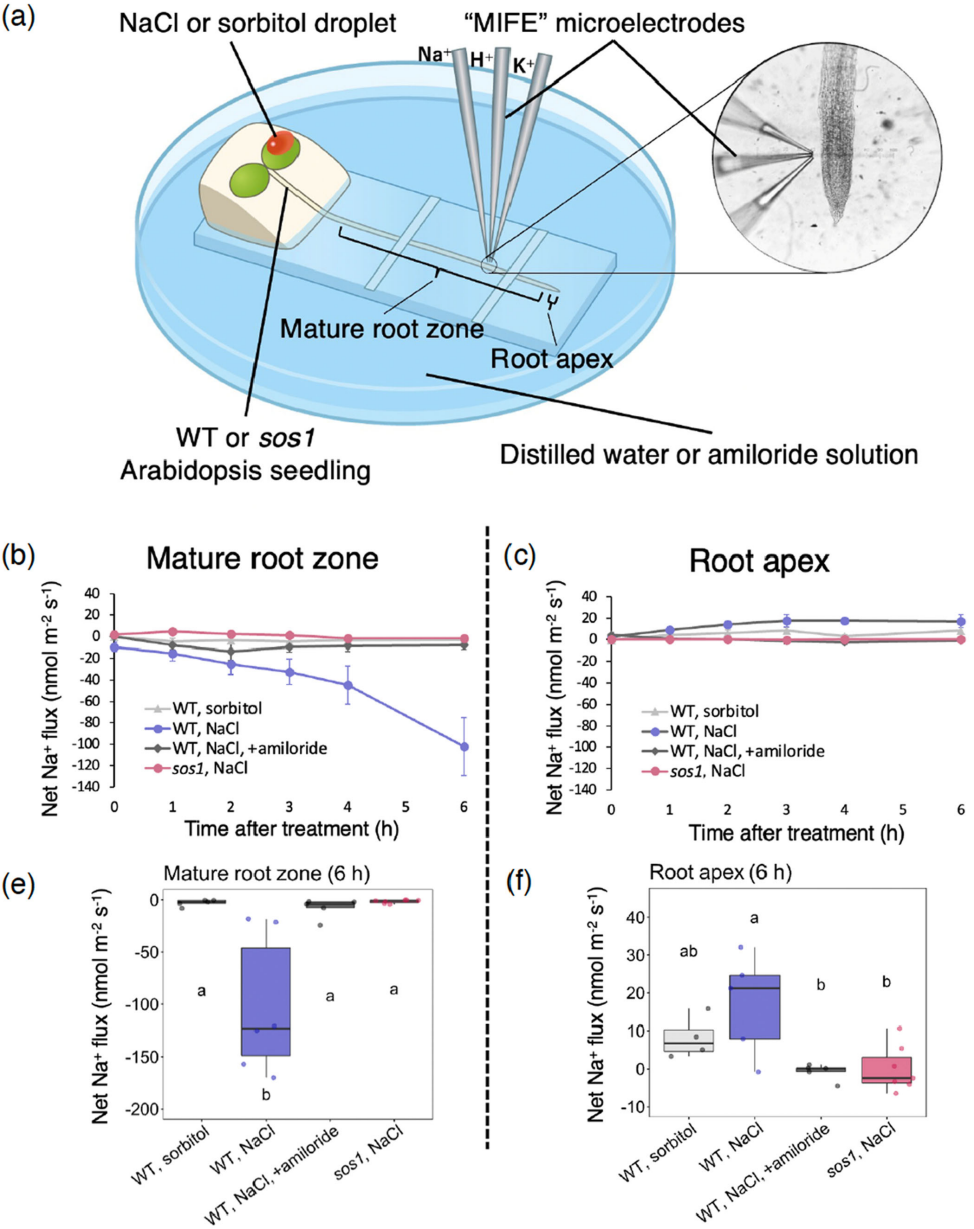
Net ion flux measurements at the root surface. (a) Schematic illustration of net ion flux measurements at the surface of an Arabidopsis root. After a 1‐h stabilization period, a droplet of 5.0 mM NaCl or isotonic 8.5 mM sorbitol solution was applied to 7‐day‐old Arabidopsis seedlings, and net ion fluxes were analysed at a specific area of the root surface. (b–f) Net Na^+^ fluxes at the surface of the mature root zone (b, e) and the root apex (c, f), approximately 5 mm and 400–600 µm from the root cap, respectively. (b, c) Time course of net Na^+^ flux before and 1, 2, 3, 4 and 6 h after foliar application. Data points and error bars in the line graph indicate the mean and standard error, respectively (*n* = 5 − 7). (e, f) Net Na^+^ flux values of 6 h after foliar application (*n* = 5–7). Different letters indicate statistically significant differences in net flux among lines or treatments (one‐way ANOVA and Tukey–Kramer's test, *p* < 0.05). [Color figure can be viewed at wileyonlinelibrary.com]

To confirm that the Na^+^ removal observed with RRIS (Figure 1b) was due to active exclusion into the rhizosphere, we employed the noninvasive Microelectrode Ion Flux Estimation (MIFE) technique to analyse ion fluxes at the root surface (Newman 2001; Shabala and Newman 1997). Consistent with the radiotracer experiments, a droplet of either NaCl or sorbitol solution was applied to an Arabidopsis leaf, and net ion fluxes were then measured at the root surface (Figure 2a). Following the foliar application of NaCl solution, Na^+^ efflux progressively increased over 6 h at the mature root zone of WT roots (Figure 2b,e), whereas no increase in net efflux was observed in *sos1* roots (Figure 2b,e). Continuous measurement of net Na^+^ flux in WT roots showed that the magnitude of Na^+^ efflux continued to increase up to 12 h (Supporting Information S1: Figure S3). No increase in Na^+^ efflux was detected when sorbitol solution, which induces the same osmotic pressure as the NaCl solution, was applied (Figure 2b–f), indicating that the increase in Na^+^ efflux was specific to NaCl and not triggered by osmotic stress. Furthermore, in the presence of amiloride, an inhibitor of Na^+^/H^+^ antiporters including SOS1 (Wu et al. 2019), WT roots did not show Na^+^ efflux (Figure 2b–f). At the root apex, WT roots exhibited net Na^+^ influx (Figure 2c,f), possibly representing reabsorption of the excluded Na^+^, given that Na^+^ was not supplied in the basal medium. Net fluxes of potassium ions (K^+^) and protons (H^+^) were measured simultaneously with Na^+^ fluxes, and no significant differences were observed between WT plants with or without Na^+^ efflux activity (Supporting Information S1: Figure S4). We further analysed the electrical potential difference across the plasma membrane at the mature root zone of WT plants. Foliar application of NaCl solution induced the hyperpolarization of root epidermal cells after 6 h (Supporting Information S1: Figure S5), which occurred following the onset of Na^+^ efflux.

### 2.3 Phloem‐Derived Na^+^ Is Predominantly Excluded via SOS1 From the Mature Root Zone Into the Rhizosphere

**Figure 3:**
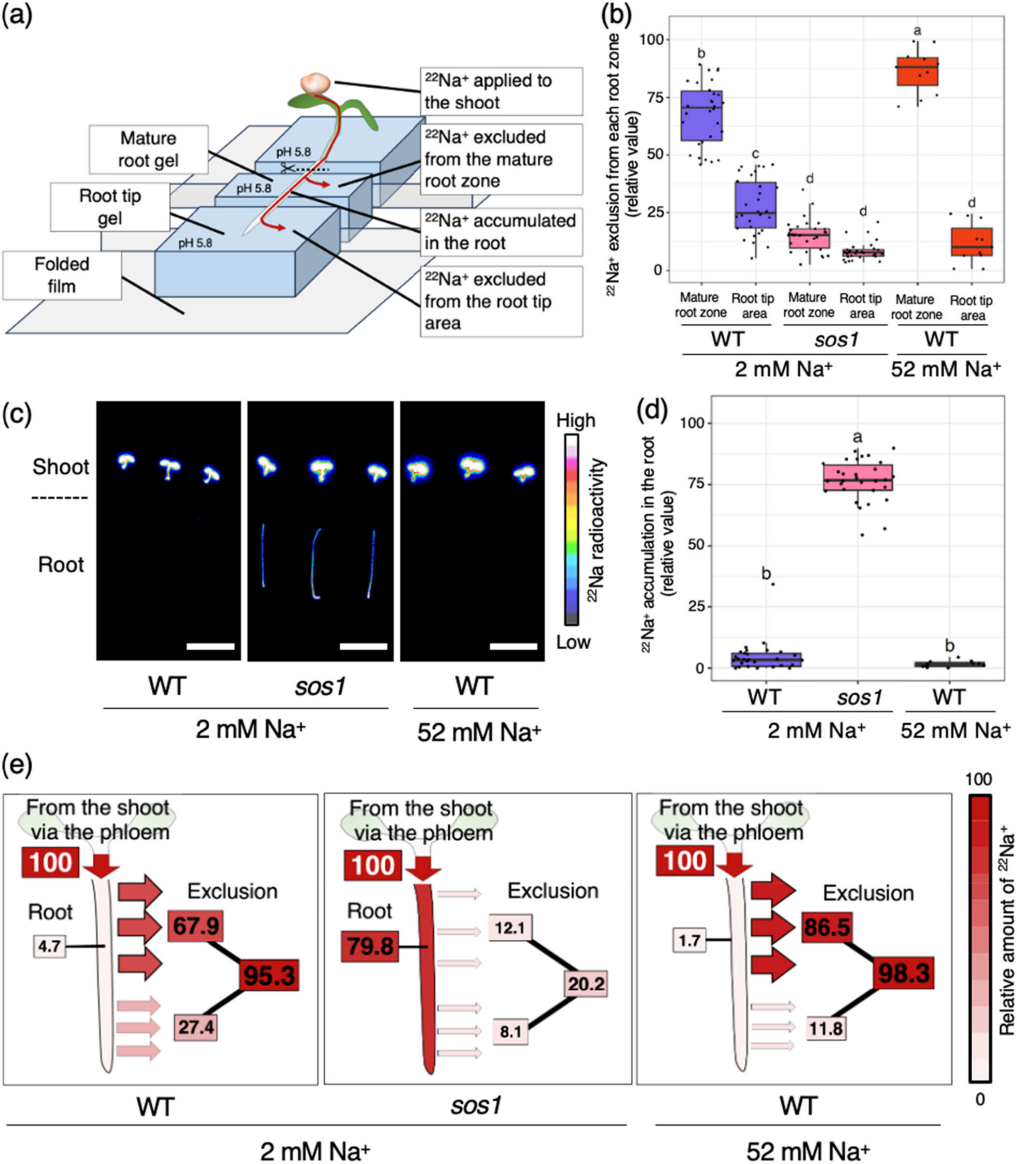
Distribution of Na^+^ transported from the shoot through the phloem in Arabidopsis WT plants and *sos1* mutants. (a) Schematic illustration of radioactive sodium ion (^22^Na^+^) tracer experiments with shoot application using the Air Gap Gel System. Plants were grown for 4 days on a nutrient medium containing 2 mM Na^+^, then transferred to medium with either 2 mM or 52 mM Na^+^ for 1 day. Then 5‐day‐old Arabidopsis seedlings were arranged on precut and aligned nutrient medium gel blocks, and ^22^Na^+^ was applied to the leaves in solution form. Twelve hours after the application, the shoot was removed from each plant, and the amount of ^22^Na^+^ was analysed in the root, the gel beneath the mature root and the gel beneath the root tip (referred to as the ‘Mature root gel’ and ‘Root tip gel’, respectively). (b) Relative amount of ^22^Na^+^ excluded from the mature root zone and the root apex. The relative amount of ^22^Na^+^ was calculated based on the total amount of ^22^Na^+^ transported from the shoot via the phloem over 12 h. Each dot represents a biological replicate (*n* = 11–30). Different letters indicate statistically significant differences (one‐way ANOVA and Tukey–Kramer's test, *p* < 0.05). (c) Representative autoradiograms showing ^22^Na^+^ accumulated in the plant samples of WT and *sos1*. Scale bars = 10 mm. (d) Relative amount of ^22^Na^+^ accumulated in the root (*n* = 11–30). (e) Distribution profile of ^22^Na^+^ applied to the leaf. Numbers represent the average percentages (*n* = 11–30) of ^22^Na^+^ distributed to each part, relative to the total amount transported from the shoot through the phloem over 12 h. [Color figure can be viewed at wileyonlinelibrary.com]

To assess the role of SOS1 in excluding phloem‐derived Na^+^ during Na^+^ recirculation—where Na^+^ is transported from leaves back to the root—we quantified ^22^Na^+^ transported into the root zone after foliar application using a radiotracer‐based method we previously developed as the Air Gap Gel System (Figure 3a; Nagata et al. 2023). Under 2 mM Na^+^ conditions, WT plants excluded a large proportion of the recirculated Na^+^ from both the mature root zone and root tip area, accounting for approximately 68% and 27% of the total Na^+^, respectively. In contrast, Na^+^ exclusion was markedly impaired in *sos1* mutants, with only 12% excluded from the mature root zone and 8% from the root tip area (Figure 3b,e). As a result, the total Na^+^ retained in *sos1* roots was significantly higher, averaging ~80%, compared to just 5% in WT roots (Figure 3c–e). To further investigate the effect of salt stress on exclusion patterns, we analysed WT plants under 52 mM Na^+^ conditions. Because *sos1* mutants exhibit severe Na^+^ toxicity and tissue damage under 52 mM Na^+^, this condition was not applied to *sos1* plants in this experiment. In this condition, Na^+^ exclusion from the mature root zone increased to 87%, whereas Na^+^ exclusion from the root tip area decreased to 12%, compared to the 2 mM Na^+^ condition (Figure 3b,e). The total Na^+^ retained in roots remained low and was comparable to that under the 2 mM Na^+^ condition (2% vs. 5%, respectively) (Figure 3c–e). These results indicate that SOS1 works in the mature root zone for phloem‐derived Na^+^ exclusion.

### 2.4 Xylem‐Derived Na^+^ Is Also Excluded From the Mature Root Zone With Partial Contribution of SOS1

**Figure 4:**
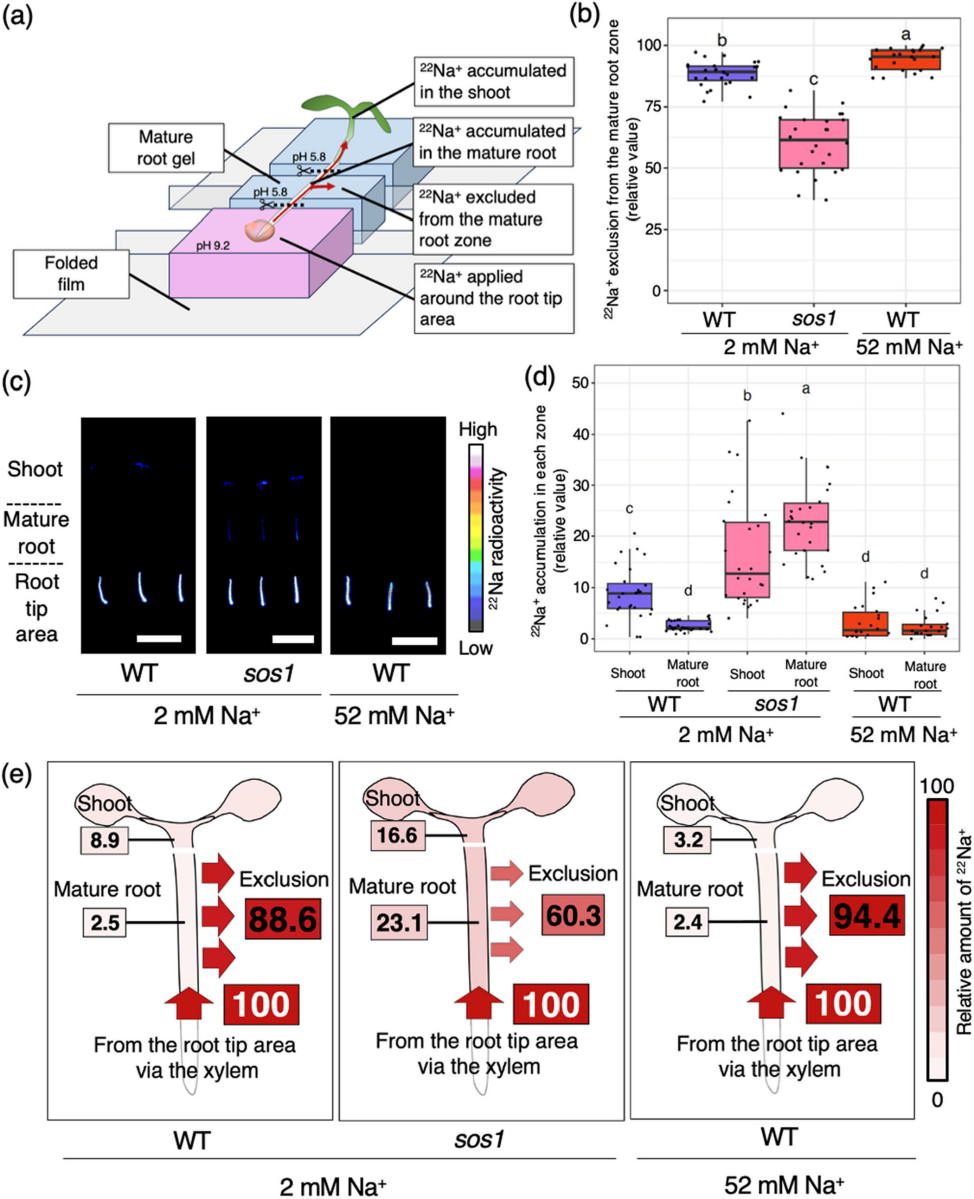
Distribution of Na^+^ transported from the root through the xylem in Arabidopsis WT plants and *sos1* mutants. (a) Schematic illustration of radioactive sodium ion (^22^Na^+^) tracer experiments with root application using the Air Gap Gel System. Plants were grown for 4 days on a nutrient medium containing 2 mM Na^+^, then transferred to medium with either 2 mM or 52 mM Na^+^ for 1 day. Then, 5‐day‐old Arabidopsis seedlings were arranged on precut and aligned nutrient medium gel blocks, and ^22^Na^+^ was applied exclusively to the root tip area in solution form. 4 h after the application, the seedlings were cut, and the amount of ^22^Na^+^ was analysed in the shoot, the mature root and the gel beneath the mature root (referred to as the ‘Mature root gel’). (b) Relative amount of ^22^Na^+^ excluded from the mature root zone. The relative amount of ^22^Na^+^ was calculated based on the total amount of ^22^Na^+^ transported from the root tip area via the xylem over 4 h (*n* = 22–27). (c) Representative autoradiograms showing ^22^Na^+^ accumulated in the plant samples of WT and *sos1*. Scale bars = 10 mm. (d) Relative amount of ^22^Na^+^ accumulated in the shoot and the mature root zone (*n* = 22–27). (e) Distribution profile of ^22^Na^+^ applied to the root tip area. Numbers represent the average percentages (*n* = 22–27) of ^22^Na^+^ distributed to each part, relative to the total amount transported from the root tip area through the xylem over 4 h. [Color figure can be viewed at wileyonlinelibrary.com]

We found that SOS1 in the mature root zone contributes to phloem‐derived Na^+^ exclusion during Na^+^ recirculation (Figure 3e). However, it remained unclear whether this function is restricted to phloem‐derived Na^+^ or also applies to Na^+^ transported via the xylem. To assess the role of SOS1 in excluding xylem‐derived Na^+^ from the mature root zone—where Na^+^ is transported upward from the root tip via the xylem—we quantified the upward transport of ^22^Na^+^ into the mature root zone and further to the shoot following application at the root tip, using a radiotracer‐based method, Air Gap Gel System (Figure 4a; Nagata et al. 2023). Under 2 mM Na^+^ conditions, 89% of the ^22^Na^+^ transported from the root tip through the xylem was subsequently excluded from the mature root zone into the surrounding gel in WT plants. In contrast, this exclusion rate was significantly reduced to 60% in *sos1* mutants (Figure 4b,e), indicating impaired exclusion. Consequently, *sos1* mutants retained substantially more Na^+^ in the mature root zone (23%) compared to WT plants (3%) (Figure 4c–e). Under 52 mM Na^+^ conditions, the exclusion rate from the mature root zone in WT plants increased significantly to 94%, while Na^+^ accumulation in the shoot decreased significantly to 3%, reaching a level comparable to that in the mature root zone (Figure 4b,d,e). These results indicate that SOS1 also works in the mature root zone for xylem‐derived Na^+^ exclusion. Same as the phloem‐derived Na^+^ experiment in Figure 3, the 52 mM Na^+^ condition was not applied to *sos1*, because *sos1* mutants exhibit severe Na^+^ toxicity and tissue damage under this condition.

## 3 Discussion

### 3.1 Mature Root Zone as the Primary Site of SOS1‐Mediated Exclusion of Recirculated Na^+^

Plants mitigate Na^+^ toxicity in shoots by returning Na^+^ back to roots via the phloem. This process, termed Na^+^ recirculation (Alfocea et al. 2000; Matsushita and Matoh 1991; Zhao et al. 2020), serves to protect photosynthetically active regions from the harmful effects of excess Na^+^. The transporter HKT1 has been implicated in retrieving Na^+^ into the phloem in shoots (Berthomieu et al. 2003); however, the fate of this phloem‐derived Na^+^ in roots has remained unclear. Among known transporters, SOS1 is the only transporter characterized to mediate Na^+^ exclusion from the intracellular to the extracellular space at the plasma membrane in roots (Zhao et al. 2020). However, its specific contribution to excluding phloem‐derived Na^+^ had not been quantified. In the present study, by using radiotracer imaging and microelectrode measurements, we provide direct visual and quantitative evidence that SOS1 is essential for excluding phloem‐derived Na^+^ from bulk roots during Na^+^ recirculation (Figures 1, 2 and 2, Supporting Information S1: Figure S1, Video S1). This provides the first direct evidence of the SOS1 involvement in the exclusion of phloem‐derived Na^+^ from roots. Furthermore, our findings indicate that about 95% of phloem‐derived Na^+^ was excluded from the root. In contrast, only ~20% was excluded in the *sos1* mutant, which retained ~80% in the root (Figure 3e). These results identify SOS1 as the predominant Na^+^ exclusion system during Na^+^ recirculation.

To date, the spatial locus of SOS1 action in roots has been debated. Promoter‐GUS analysis reported strong activity in the epidermis at the root apex and weaker activity in xylem parenchyma within the endodermal barrier, suggesting a role at the apex (Shi et al. 2002). Conversely, a cell‐type‐specific microarray analysis and a single‐cell RNA‐Seq analysis indicated broad SOS1 expression across root cell types, including the outer layers of the mature root zone (Brady et al. 2007; Ryu et al. 2019). Our radiotracer experiments resolve this at the whole‐plant level: SOS1‐mediated exclusion of phloem‐derived Na^+^ occurs predominantly in the mature root zone and is the major contributor to Na^+^ efflux from the root to the rhizosphere (Figure 3e). Functionally, prioritizing exclusion in the basal mature regions would prevent recirculated Na^+^ from reaching the elongation zone at the apex—an area particularly sensitive to salinity (Oh et al. 2009). Given that overall Na^+^ tolerance correlates with root Na^+^ exclusion capacity (Lessani and Marschner 1978; see also Introduction), and that the mature root zone constitutes most of the root system, it is plausible to conclude that the mature root zone is the principal site of Na^+^ exclusion.

We also quantified the contribution of SOS1 to the exclusion of xylem‐derived Na^+^ (Figure 4). Notably, SOS1 had a stronger impact on the exclusion of phloem‐derived Na^+^ than xylem‐derived Na^+^ (Figures 3, 4 and 4), consistent with its role as a plasma‐membrane Na^+^ efflux transporter. Phloem‐derived Na^+^ is likely to enter the cytosol of root cells during Na^+^ recirculation, making SOS1 particularly influential in this process. Our radiotracer experiments using the air‐gap gel system also revealed additional Na^+^ efflux mechanisms independent of SOS1. To date, no such SOS1‐independent pathways have been reported, highlighting the need for further investigation to elucidate this novel mechanism.

Although SOS1 is expressed in the shoot (Brady et al. 2007; Shi et al. 2002), foliar Na^+^ application still resulted in Na^+^ arrival at the roots of *sos1* mutants (Figures 1, 3 and 3, Supporting Information S1: Figure S1, Video S1), indicating that SOS1 is not required for loading shoot‐derived Na^+^ into the phloem during Na^+^ recirculation. As previously described, HKT1 mediates Na^+^ loading into the phloem to facilitate leaf Na^+^ clearance (Berthomieu et al. 2003; Sunarpi et al. 2005). Taken together, our data support a model in which HKT1 mediates the recirculation step itself, whereas SOS1 acts downstream in the root to exclude the incoming phloem‐derived Na^+^. Once delivered to the root, phloem‐derived Na^+^ appears to follow two principal fates—rapid exclusion to the rhizosphere or sequestration within root cells—and a fraction of the excluded Na^+^ may be reloaded into the xylem and returned to the shoot. Quantifying the relative fluxes through these routes, including possible xylem reloading, remains an important objective for future study.

Recent studies have suggested a molecular link between HKT1 and the SOS pathway (e.g., Gámez‑Arjona et al. 2024). Although these pathways have often been studied separately, their functions may be coordinated. Specifically, Na^+^ recirculated to the roots via HKT1 could be actively excluded by SOS1, forming a two‐step Na^+^ detoxification system. This coordinated mechanism may play a central role in enhancing plant salt tolerance.

### 3.2 Salt‐Concentration‐Dependent SOS1‐Mediated Na^+^ Exclusion

Activation of SOS1 through the SOS pathway is essential for root Na^+^ exclusion. Mechanistically, SOS1 is activated when its autoinhibitory domain is phosphorylated by SOS2/CIPK24 (Jarvis et al. 2014; Quintero et al. 2011). This phosphorylation is triggered by Ca^2^^+^ sensors such as SOS3/CBL4 and SCaBP8 (Halfter et al. 1999; Kim et al. 2007; Quan et al. 2007). Salt‐evoked elevations in cytosolic Ca^2+^—potentially initiated by Na^+^‐GIPC interactions followed by Ca^2+^ influx (Jiang et al. 2019; Knight et al. 1997) —likely underlie this response. The fact that SOS1‐mediated Na^+^ exclusion operates under mild salt stress (2 mM Na^+^ condition) suggests that either modest increases in cytosolic Na^+^ are sufficient to trigger a Ca^2+^ signal, or that a basal level of SOS pathway activity is enough to sustain Na^+^ exclusion under such conditions. Notably, previous studies have shown that Ca^2+^ influx is typically observed at ~50–200 mM NaCl (Jiang et al. 2019; Steinhorst et al. 2022), and that SOS2‐mediated phosphorylation of SOS1's autoinhibitory domain is usually observed at ~100 mM NaCl (Quintero et al. 2011), implying that the SOS1 activity detected here occurs at Na^+^ levels lower than those generally required for strong SOS pathway activation. At higher salinity (75–200 mM Na^+^), additional modules such as CBL8–CIPK24 have been reported to activate SOS1 (Steinhorst et al. 2022), suggesting multiple Na^+^‐dependent regulatory mechanisms. Consistent with these findings, under the 52 mM Na^+^ condition, the exclusion of both phloem‐ and xylem‐derived Na^+^ from the mature root zone increased relative to the 2 mM Na^+^ condition (Figures 3 and 4). At the same time, Na^+^ distribution shifted: under the 2 mM Na^+^ condition, it was biased toward the shoot, whereas under the 52 mM Na^+^ condition, it was present at comparable levels in the shoot and mature root (Figure 4). HKT1 activity may contribute to this shift (Berthomieu et al. 2003; Sunarpi et al. 2005), although the mechanism remains to be resolved. A limitation is that 52 mM Na^+^ induces excessive Na^+^ accumulation and severe damage in *sos1* mutants, complicating the isolation of exclusion phenotypes from secondary effects (e.g., membrane disruption and ion leakage). To accurately assess SOS1‐dependent Na^+^ exclusion under severe salinity, it will be necessary to also monitor cell integrity and ion homeostasis.

### 3.3 Summary

Using radiotracer and MIFE techniques, we characterized Na^+^ dynamics derived from root‐to‐shoot transport and phloem‐mediated recirculation. Our findings highlight that SOS1 plays a significant role in Na^+^ exclusion in the mature root zone, where the majority of phloem‐derived Na^+^ is excluded to the rhizosphere.

## 4 Materials and Methods

### 4.1 Plant Materials and Growth Conditions

Seeds of *Arabidopsis thaliana* ecotype Columbia‐0 (Inplanta Innovations Inc., Yokohama, Japan) and T‐DNA insertion mutant line of *AtSOS1* (CS3862; Arabidopsis Biological Resource Center, Columbus, OH, USA) were surface sterilized with 1% sodium hypochlorite (FUJIFILM Wako Pure Chemical, Osaka, Japan) and 0.02% Triton X‐100 (FUJIFILM Wako Pure Chemical, Osaka, Japan) and rinsed thoroughly with distilled water. For radiotracer experiments, plants were grown on MGRL medium (Fujiwara et al. 1992) solidified with 0.4% gellan gum (FUJIFILM Wako Pure Chemical, Osaka, Japan) at 22°C under a 16 h light (100 µmol m^−2^ s^−1^) and 8 h darkness photoperiod for 5 or 7 days. For ion flux analyses, plants were grown under the same conditions for 7 days, except that the growth medium was modified. To exclude Na^+^ from the medium, 1.5 mM NaH₂PO₄ and 0.25 mM Na_2_HPO_4_ in the original MGRL medium were replaced with 1.75 mM (NH_4_)_2_PO_4_. Additionally, 67 µM EDTA‐2Na and 8.6 µM FeSO_4_ were replaced with 65 µM Fe(III)‐EDTA (equivalent to 8.6 µM Fe^3^^+^). To maintain the pH at 5.8, approximately 250 µM KOH was added, because the phosphate buffer present in the original medium was removed.

### 4.2 Sequential Radioisotope Imaging of Phloem‐Derived ^22^Na^+^ in Roots

To visualize phloem‐derived Na^+^, we used the RRIS (Nakanishi et al. 2009). Seven‐day‐old Arabidopsis seedlings were transferred to fresh solid growth medium, with the shoots positioned over the edge of the medium. To bring the roots into contact with a fibre optic plate with a scintillator (FOS), each plant was placed in a 10‐µm‐thick polyethylene pocket with an open top. The pocket was then placed on the FOS so that the roots were sandwiched between the medium and the FOS (Figure 1a). A 1‐μL droplet of radioisotope solution was applied to one cotyledon. Imaging began immediately after applications, inside a dark box. Images were captured hourly by a CCD camera (C3077‐70, Hamamatsu Photonics Co., Shizuoka, Japan) for 15 min in darkness, followed by 45 min of illumination, as previously described by Hirose et al. (2013). This imaging‐illumination cycle was repeated for up to 24 h after isotope application. The radioisotope and carrier ion concentrations in the applied solutions were as follows: ^22^Na^+^, 3.5 kBq μL^−^^1^ and 0.5 mM (^22^Na^+^ purchased from Eckert & Ziegler, Valencia, CA, USA); ^42^K^+^, 4 kBq μL⁻¹ and 1 mM (^42^K^+^ was generated using a ^42^Ar–^42^K generator; Aramaki et al. 2015). Because ^42^K has a short half‐life of 12.36 h, images obtained with ^42^K were corrected for radioactive decay using its half‐life in ImageJ.

### 4.3 Determination of Net Ion Flux in Roots

Net ion fluxes at the root surface were measured non‐invasively with ion‐selective microelectrodes (MIFE technique; Newman 2001; Shabala and Newman 1997). Ion‐selective microelectrodes and the reference electrode were prepared as previously described (Shabala and Newman 1997). For the preparation of Na^+^‐, K^+^‐ and H^+^‐selective microelectrodes, the microelectrodes were backfilled with 500 mM NaCl, 200 mM KCl, and a mixture of 15 mM NaCl and 40 mM KH₂PO₄, respectively. The Na^+^‐selective microelectrodes were front‐filled with an improved calixarene‐based Na^+^ ionophore cocktail (Jayakannan et al. 2011), and K^+^‐ and H^+^‐selective microelectrodes were front‐filled with commercially available ionophore cocktails (product numbers 60031 and 95297, respectively; Sigma‐Aldrich, St. Louis, MO, USA). Three microelectrodes were mounted on a 3D‐micromanipulator (MMT‐5, Narishige, Tokyo, Japan), and calibrated using sets of three solutions with different ion concentrations (10, 20, 50 µM NaCl for Na^+^; 10, 20, 50 µM KCl for K^+^; pH 5.1, 6.4, 7.8 for H^+^). Only electrodes with a Nernstian slope of more than 50 mV/decade and a correlation of more than 0.999 were used. The tips of the microelectrodes were positioned close together and placed 40 µM above the root surface. A computer‐controlled stepper motor moved the microelectrodes every 6 s (or every 30 s for continuous measurements) between two positions: 40 and 150 µM from the root surface. The potential difference between these two positions was recorded, and the net ion fluxes were calculated as previously described (Newman 2001). For continuous measurements, average net flux values were calculated every 5 min.

To localize the site of Na^+^ exclusion, Na^+^ flux was measured at the root apex (400–600 µm from the root cap) and the mature root zone (5 mm from the root cap) of Arabidopsis WT and *sos1* mutant seedlings. Seven‐day‐old seedlings were immobilized on microscope slides using strips of paraffin film. Roots were pretreated with distilled water or 100 µM amiloride solution (both with 0 µM Na^+^), with the shoot kept above the solution. To stabilize root physiological activity after potential disturbances caused by immobilization, the immobilized roots were kept in the pretreatment solution for 1 h. Before the application of solutions to a cotyledon, steady‐state net fluxes of Na^+^, K^+^ and H^+^ were measured at both the root apex and the mature root zone for 3–5 min to ensure stable initial values. A 5‐µL droplet of either a 5 mM NaCl or an 8.5 mM sorbitol solution was applied to a cotyledon, and net ion fluxes from the two zones were measured hourly for up to 6 h after the application. Each condition and line were tested with 5–7 biological replicates.

### 4.4 Determination of Membrane Potential

The electrical potential difference across the plasma membrane was determined by measuring electrical potentials inside and outside the plasma membrane using a microelectrode as previously described (Shabala and Newman 1997). For the microelectrode preparation, a borosilicate glass capillary with an internal filament (GC150F‐10, Harvard Apparatus, Cambridge, MA, USA) was pulled and filled with a 500 mM KCl solution. A reference electrode was prepared in the same manner as for the net ion flux analysis. Seven‐day‐old Arabidopsis WT seedlings were immobilized on plastic blocks using strips of paraffin film, and their roots were kept in 10 mL of distilled water for 1 h. A 5‐µL droplet of 5 mM NaCl solution was then applied to one cotyledon. Electrical potential in epidermal root cells was measured by impaling the cells with a microelectrode. Membrane potential was measured at three time points: before the NaCl application, and 1 and 6 h after the application. Average membrane potential values were calculated from 2 to 6 impalements made for each seedling. Four to six seedlings were tested for each condition.

### 4.5 Measurement of Root Na^+^ Exclusion Using the Air Gap Gel System

To evaluate Na^+^ exclusion from roots, we used the Air Gap Gel System combined with the radioisotope ^22^Na, as described by Nagata et al. (2023). Briefly, solid MGRL medium gel blocks (0.6% gellan gum) were aligned with approximately 1 mm air gaps, as shown in Figures 3a and 4a. Five‐day‐old Arabidopsis seedlings were arranged on these precut and aligned MGRL gel blocks. The seedlings were first grown for 4 days on standard MGRL medium containing 2 mM Na^+^ and then transferred to either standard MGRL medium with 2 mM Na^+^ (2 mM Na^+^ condition) or MGRL medium containing 52 mM Na^+^ (52 mM Na^+^ condition) for 1 additional day. The solid MGRL medium gel blocks also contained either 2 mM or 52 mM Na^+^, depending on the Na^+^ condition.

For phloem‐derived ^22^Na^+^ analysis (Figure 3a), 1 μL drop of ^22^Na^+^ solution (3.7 kBq ^22^Na^+^ in either 2 mM or 52 mM Na^+^, depending on the Na^+^ condition) was applied to the leaf surface. After 12 h, the gel blocks and plants were collected.

For xylem‐derived ^22^Na^+^ analysis (Figure 4a), the pH of the gel blocks surrounding the root apex was adjusted to 9.2 using 2‐amino‐2‐hydroxymethyl‐1,3‐propanediol (Tris; FUJIFILM Wako Pure Chemical, Osaka, Japan) to block SOS1 activity and promote Na^+^ uptake (Nagata et al. 2023). A 1 μL drop of the same solution was applied around the root apex, and samples were collected after 4 h. To minimize the potential influence of phloem‐derived Na^+^, the incubation time for the xylem‐derived Na^+^ transport assay was limited to 4 h, whereas a longer duration (12 h) was applied in the phloem‐derived Na^+^ experiment.

The ^22^Na^+^ retained in the gel blocks was extracted using pure water and mixed with Ultima Gold scintillation cocktail (PerkinElmer, Waltham, MA, USA). ^22^Na activity was measured using a liquid scintillation counter (TriCarb 4810 TR; PerkinElmer). ^22^Na signals in plant tissues were visualized using an imaging plate and an Imaging Analyser (Amersham Typhoon; GE Healthcare), and image analysis was performed using ImageJ software (version 1.53t).

### 4.6 Statistical Analysis

Statistical analyses were conducted using R software (version 4.1.1). A factorial ANOVA was performed to evaluate differences in relative Na^+^ content, net Na^+^ flux and changes in membrane potential of root epidermal cells over different time points. When significant differences were found, Tukey–Kramer's test was used for multiple pairwise comparisons to identify statistically significant differences.

## Supporting information


**Supplemental Figure S1:** Foliar‐applied Na⁺ accumulation in roots of individual plants quantified from the Real‐Time Radioisotope Imaging System images. **Supplemental Figure S2:** Radioisotope imaging visualizes the transport of various elements via the phloem. **Supplemental Figure S3:** Continuous measurement of net Na^+^ flux at the mature root zone. **Supplemental Figure S4:** Sodium‐ion exclusion is not accompanied by changes in net potassium ion flux or net proton flux. **Supplemental Figure S5:** Sodium‐ion exclusion is accompanied by hyperpolarization of root epidermal cells.

Video1.

Video2.

## Data Availability

The data that support the findings of this study are available from the corresponding author upon reasonable request.
