# Supplementary material for: SALT OVERLY SENSITIVE 1 Na+/H+ Exchanger Operates in Mature Root Zone and Is a Major Contributor to Root Na+ Exclusion During Shoot‐to‐Root Na+ Recirculation
Source: Plant Cell Environ. 2025 Dec 12;49(3):1650–60. doi: 10.1111/pce.70317 (PMC12873520; doi:10.1111/pce.70317)
Supplement: Supplementary file 1 — Supplemental Figure S1: Foliar‐applied Na⁺ accumulation in roots of individual plants quantified from the Real‐Time Radioisotope Imaging System images. Supplemental Figure S2: Radioisotope imaging visualizes the transport of various elements via the phloem. Supplemental Figure S3: Continuous measurement of net Na+ flux at the mature root zone. Supplemental Figure S4: Sodium‐ion exclusion is not accompanied by changes in net potassium ion flux or net proton flux. Supplemental Figure S5: Sodium‐ion exclusion is accompanied by hyperpolarization of root epidermal cells. [file PCE-49-1650-s002.docx]

**SALT OVERLY SENSITIVE 1 Na^+^/H^+^ exchanger operates in mature root zone and is a major contributor to root Na⁺ exclusion during shoot-to-root Na⁺ recirculation**

Tomoki Nagata^1†^, Ryohei Sugita^1,2†^, Takaaki Ogura^1,3†^, Mio Nagoya^1^, Natsuko I. Kobayashi^1^, Muhammad B. Gill^3,4^, Lana Shabala^3,4,5^, Tomoko M. Nakanishi^1^, Sergey Shabala^3,4,5^, and Keitaro Tanoi^1,6*^

^1^Graduate School of Agricultural and Life Sciences, The University of Tokyo, Bunkyo-ku, Tokyo, 113-8657 Japan

^2^Radioisotope Research Center, Nagoya University, Nagoya, Aichi 464-8602, Japan

^3^Tasmanian Institute of Agriculture, College of Science and Engineering, University of Tasmania, Hobart, TAS 7005, Australia

^4^International Research Centre for Environmental Membrane Biology, Foshan University, Foshan 528000, China

^5^School of Biological Science, University of Western Australia, Perth, WA 6009, Australia

^6^Fukushima Institute for Research, Education and Innovation, Yazawa-machi, Gongendo, Namie town, Futaba County, Fukushima 979-1521, Japan

^†^These authors contributed equally to this work

*Correspondence to: uktanoi@g.ecc.u-tokyo.ac.jp

**Supplemental data**


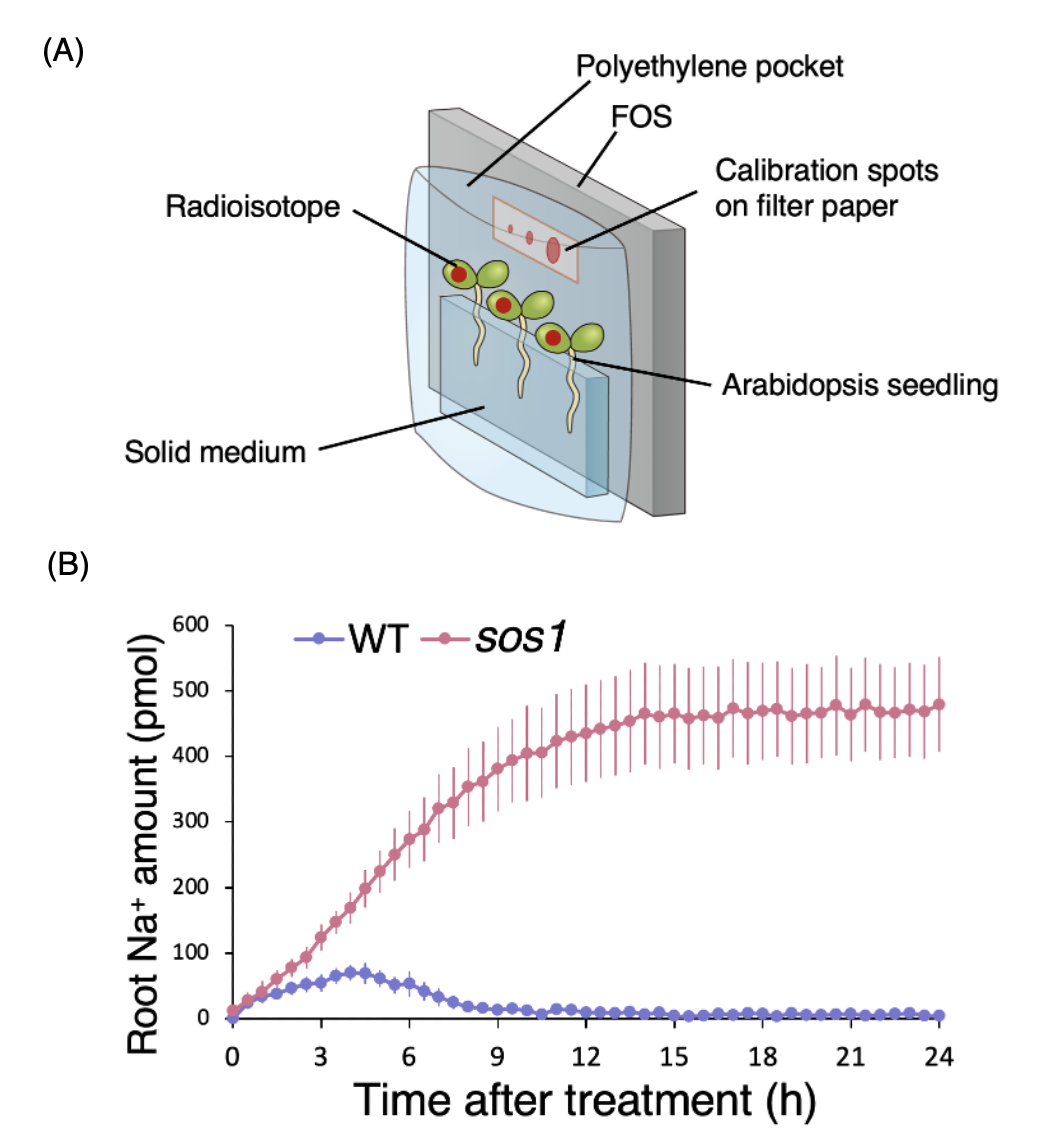


**Supplemental Figure S1. Foliar-applied Na⁺ accumulation in roots of individual plants quantified from the Real-Time Radioisotope Imaging System images.**

(A) Schematic illustration of the method used to quantify Na⁺ concentration with the Real-Time Radioisotope Imaging System (RRIS). The experiment was conducted under the same conditions as Fig. 1b, and Na⁺ content was calculated based on the calibration spots obtained from the acquired data. (B) Time course of root Na⁺ accumulation after foliar Na⁺ application. Data points and error bars in the line graph indicate the mean and standard error, respectively (n = 5).


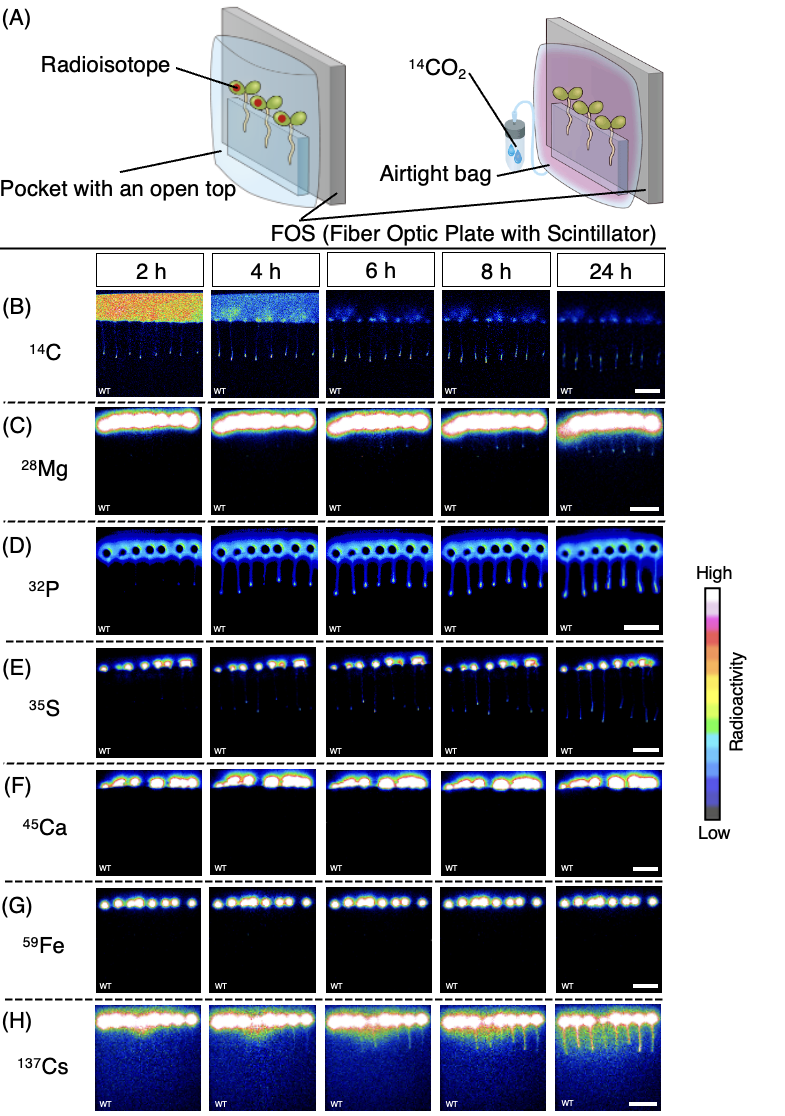


**Supplemental Figure S2. Radioisotope imaging visualizes the transport of various elements via the phloem.**

(A) Schematic diagram illustrating the application of radioisotopes to plants using the Real-time Radioisotope Imaging System (RRIS). Seven-day-old Arabidopsis seedlings were placed on solid medium, and radioisotopes were applied to the leaves either in solution form or in gaseous form (^14^CO_2_). Radiation emitted from the radioisotopes was converted into visible light and captured by a CCD camera to obtain images.

(B) RRIS images of ^14^C, (C) ^28^Mg, (D) ^32^P, (E) ^35^S, (F) ^45^Ca, (G) ^59^Fe, and (H) ^137^Cs. Scale bars = 20 mm.


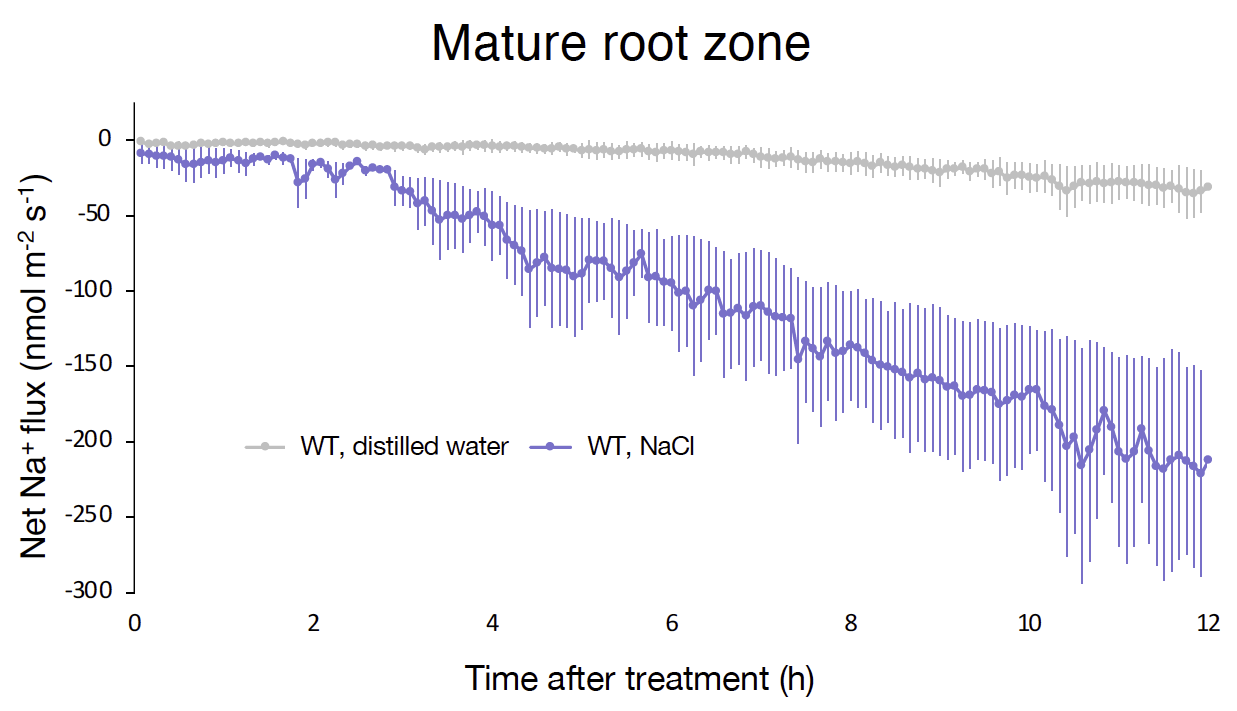


**Supplemental Figure S3. Continuous measurement of net Na^+^ flux at the mature root zone.**

Net Na^+^ flux from the surface of the mature root zone (5 mm from the root cap) after foliar application of 5 mM NaCl solution (red) or water (grey) to seven-day-old Arabidopsis wild-type seedlings. Data points and error bars in the line graph indicate the mean and standard error, respectively (NaCl solution, n = 3; water, n = 2).


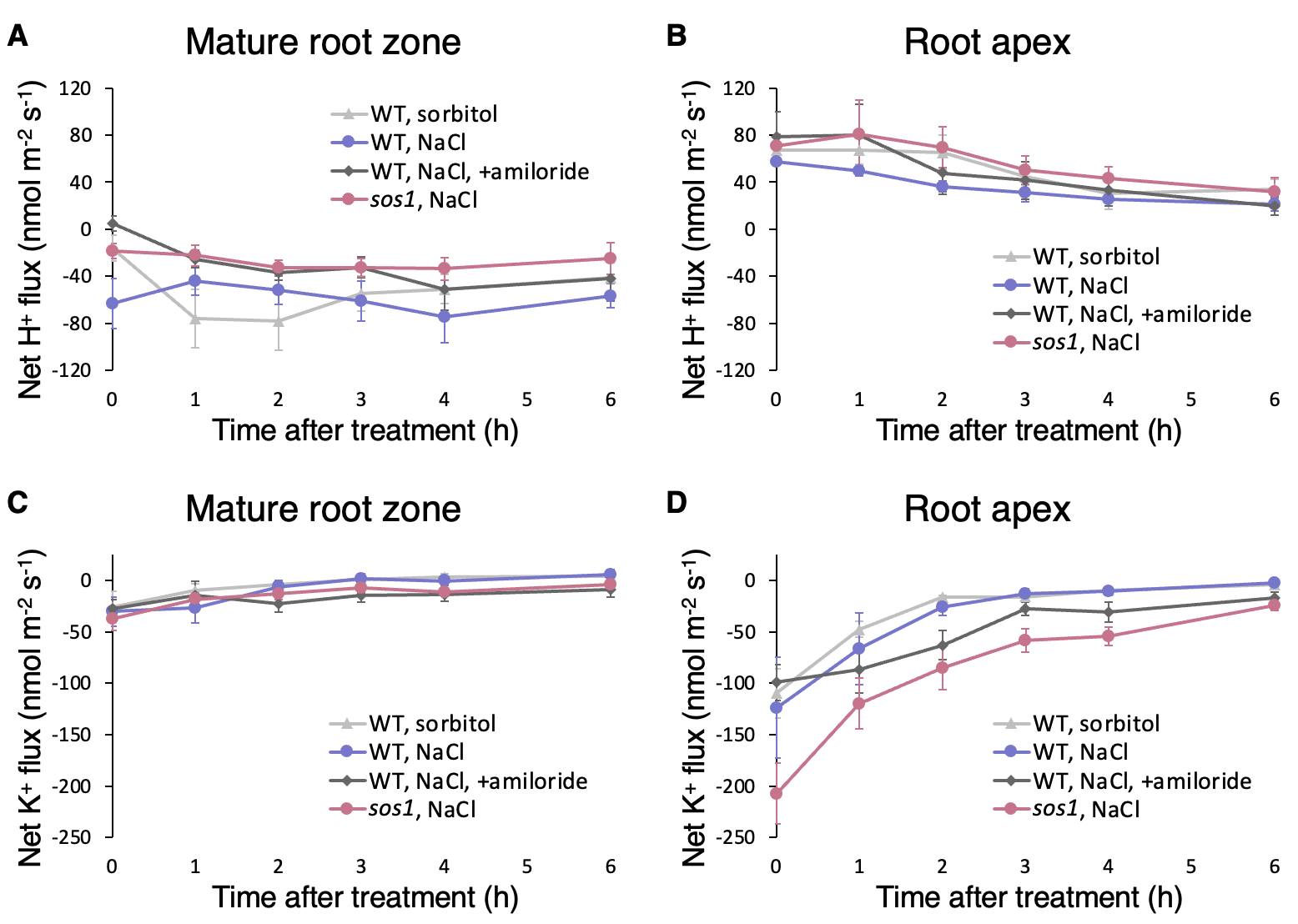


**Supplemental Figure S4. Sodium-ion exclusion is not accompanied by changes in net potassium ion flux or net proton flux.**

(**A**-**D**) Net K^+^ flux (**A**, **B**) and net H^+^ flux (**C**, **D**) from the surface of the mature root zone (**A**, **C**; 5 mm from the root cap) and root apex (**B**, **D**; 400 – 600 µm from the root cap) of seven-day-old Arabidopsis seedlings after the foliar application of 8.5 mM sorbitol solutions to the wild type (WT), 5 mM NaCl solutions to WT, 5 mM NaCl solutions to WT treated with 100 µM amiloride solution, and 5 mM NaCl solutions to *SOS1* knockout mutant (*sos1*). Data points and error bars in the line graph indicate the mean and standard error, respectively (n = 5 – 8).


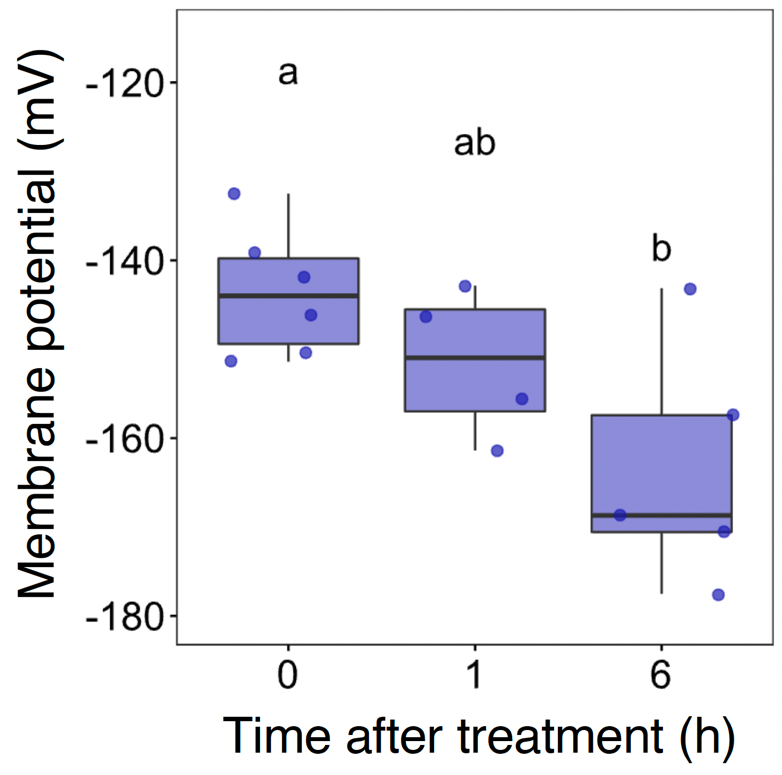


**Supplemental Figure S5. Sodium-ion exclusion is accompanied by hyperpolarization of root epidermal cells.**

The membrane potential of epidermal cells in mature root zone after the foliar application of 5 mM NaCl solutions to 7-day-old Arabidopsis wild-type seedlings. Membrane potential was measured before, 1 h after, and 6 h after the foliar application. The average membrane potential value calculated from repeated measurements in each replicate is represented as a dot (n = 4 – 6). Different letters indicate a significant difference (one-way ANOVA and Tukey Kramer’s test, *P* < 0.05).
